# Gender differences in structural and attitudinal barriers to mental healthcare in UK Armed Forces personnel and veterans with self-reported mental health problems

**DOI:** 10.1007/s00127-023-02567-0

**Published:** 2023-10-19

**Authors:** Nora Trompeter, Laura Rafferty, Daniel Dyball, Amber McKenzie, Neil Greenberg, Nicola T. Fear, Sharon A. M. Stevelink

**Affiliations:** 1 Institute of Psychiatry, Psychology and Neuroscience, King’s Centre for Military Health Research, King’s College London, 10 Cutcombe Road, London, SE5 9RJ UK; 2 Academic Department of Military Mental Health, King’s College London, London, UK

**Keywords:** Stigma, Barriers to care, Military personnel, Mental health problems, Gender differences

## Abstract

**Purpose:**

Structural and attitudinal barriers often hinder treatment-seeking for mental health problems among members of the Armed Forces. However, little is known about potential gender differences in structural and attitudinal barriers among members of the UK Armed Forces. The current study aimed to explore how men and women differ in terms of these barriers to care among a sample of UK Armed Forces personnel and veterans with self-reported mental health problems.

**Methods:**

Currently serving and ex-serving members of the UK Armed Forces who self-reported a mental health problem were invited to participate in a semi-structured phone interview on mental health and treatment-seeking. The final sample included 1448 participants (1229 men and 219 women). All participants reported on their current mental health, public stigma, self-stigma, and barriers to mental healthcare.

**Results:**

Overall, men and women reported similar levels of both structural and attitudinal barriers, with no significant differences detected. The highest scores for both men and women were observed in attitudinal barriers relating to self-stigma domains, which encapsulate internalised attitudes and beliefs about mental illness and treatment.

**Conclusions:**

Findings suggest that anti-stigma campaigns can be targeted simultaneously at both men and women within the Armed Forces. In particular, targeting self-stigma may be beneficial for health promotion campaigns.

## Introduction

Treatment-seeking for mental health problems among military personnel is often hindered due to attitudinal barriers (e.g., stigma) and structural barriers (e.g., access to mental healthcare) [1, 2]. Indeed, earlier research from the King’s Centre for Military Health Research Health and Wellbeing Cohort Study found that over one-third of UK Armed Forces personnel endorse concerns about losing military credibility and trust if they report mental health problems [3]. While such barriers are well-researched in the military population, research on how gender may impact barriers is lacking. Given the strong history of masculinity and the continued male-dominant culture of the armed forces, women may face unique barriers to receiving mental healthcare [2, 4]. Thus, the current study aimed to explore gender differences in both attitudinal and structural barriers among a sample of UK Armed Forces personnel and veterans with self-reported mental health problems.

Stigma broadly refers to the devaluation of characteristics in a particular social context [5], for example, devaluing individuals with mental health problems. This paper focuses on two distinct types of stigma: self-stigma and public stigma [6]. Public stigma describes fears of judgment from others for either having a mental illness or seeking mental health treatment [6]. This includes fear of being discriminated against, labelled, or stereotyped due to mental illness or seeking treatment for a mental health problem. Self-stigma describes the internalization of broader societal or cultural stigma of either having a mental illness or seeking mental health treatment [7]. This includes stigmatising beliefs about mental health treatment-seeking, such as feeling weak if seeking help for a mental health problem. While related, the two concepts have shown differential associations with treatment-seeking [8]. Indeed, while most studies among military samples have examined public stigma with mixed findings, self-stigma appears to have stronger associations with a lower likelihood of treatment-seeking in the US military [9].

Gender differences in stigma have been reported within the general population, with men reporting higher levels of stigma compared to women [10]. Such differences are mostly attributed to societal gender roles that emphasise care and help-seeking among women and self-reliance and toughness among men [11]. Despite an increase in women joining the military [12], military culture remains male-dominated and favours characteristics stereotypically associated with masculinity (e.g., strength, assertiveness) while devaluing characteristics stereotypically associated with femininity (e.g., caretaking, timidness; [13]). Similarly, gender differences have been reported in structural barriers, for example, women in the general population report more structural barriers related to family responsibilities (e.g., lack of childcare) compared to men [14, 15]. However, this remains to be explored within the Armed Forces.

Initial findings comparing general stigma and access to care between men and women found no significant differences for UK veterans [16]. Similarly, among US veterans, little difference was found between men and women, except that men were more likely to endorse that “*mental healthcare does not work*” compared to women, suggesting differences in stigma around mental health treatment [17]. In particular, emerging evidence suggests that women in the military may be exposed to particular types of stigma due to systemic perceptions of weakness embedded in military culture and based on masculine-ideals. For example, qualitative research among UK veterans showed that women reported a double-stigma of being seen as weak due to both experiencing mental health problems and being female [16]. However, these studies were limited by a small sample size and lack of differentiation between different types of stigma.

The current study aimed to examine potential differences in stigma and access to care between men and women in the UK Armed Forces. No a-priori hypotheses were made, as there was no clear indication from the literature whether stigma and access to care would differ between men and women in the Armed Forces.

## Methods

### Participants and procedure

Secondary data analysis using data from an interview study [18] nested within phase three of the King’s Centre for Military Health Research Health and Wellbeing Cohort Study [19] were conducted. In short, participants were sampled into the interview study from phase three of the King’s Centre for Military Health Research Health and Wellbeing Cohort Study based on self-reporting a mental health or stress problem within the last three years and subsequently were invited to complete a structured telephone clinical interview covering measures of mental health symptomatology (e.g., depression, anxiety, post-traumatic stress disorder [PTSD], and alcohol misuse), stigma, barriers to care, and help-seeking behaviour associated with their mental health. Participants received £25 for their participation. Data collection took place between February 2015 and December 2016. Further details on data collection procedures have been described elsewhere [18]. The study received ethical approval from the UK Ministry of Defence Research Ethics Committee (ref: 535/MODREC/14). The final sample included 1448 serving and ex-serving members of the UK Armed Forces. Of these, 1229 (84.9%) were men and 219 (15.1%) were women.

An a-priori power analysis using G*power [20] determined that a total sample of 496 participants would be required given the observed difference in group size (85/15) to detect gender differences in stigma scores with a small effect size (*d* = 0.30) with 80% power using a two-sample *t*-test, suggesting the current study was sufficiently powered to detect potential gender differences in barriers to mental healthcare.

### Measures

#### Stigma and access to care

Participants completed questions from the Perceived Stigma and Barriers to Care for Psychological Problems– Stigma Subscale (PSBCP-SS; [21]), the Barriers to Access Care Evaluation measure (BACE; [22]), and the Self-Stigma of Seeking Help Scale (SSSHS; [23]). Participants responded to statements relating to (i) *access to mental health services* (4 items from the PSBCP-SS), (ii) *self-stigma of mental illness* (7 items from the PSBCP-SS and BACE), (iii) *self-stigma of mental health treatment* (5 items from the SSSHS), and (iv) *public stigma of mental health care/providers* (9 items from the PSBCP-SS and BACE). Each item was rated using a 5-point response scale that ranged from (1 = *strongly agree* to 5 = *strongly disagree*). All items were reverse-scored. Scores on each subscale were averaged with higher scores reflecting greater stigma/lower access. Similar scoring approaches have been used in previous research [24]. Additionally, we used an item-response-theory (IRT) approach to generate total scores using a response style model to capture latent variables, as IRT scoring approaches have been found to perform better than mean scores when using Likert-style responses [25].

#### Mental health problems

To adjust for co-occurring mental health problems, we included information on the participant’s current mental health. This included questions on depression (Patient Health Questionnaire [PHQ-9; [26]]), generalized anxiety disorder (GAD-7 scale; [27]), post-traumatic stress disorder (20-item PTSD Checklist [PCL-5; [28]]), and alcohol misuse (3-item Alcohol Use Disorders Identification Test [AUDIT-C; [29]]). In line with previous work [18], we used the following cut-offs to indicate the presence of a probable disorder: a cut-off score of 15 on the PHQ-9 was used to identify probable depression (scores range from 0 to 27), a cut-off score of 10 or greater on the GAD-7 was used to identify probable generalised anxiety disorder (scores range from 0 to 21), a score of 38 or greater on the PCL-5 was used to identify probable PTSD (scores ranged from 0 to 78), and an AUDIT-C score of 10 or more was used to indicate alcohol misuse (scores ranged from 0 to 12).

#### Demographic variables

Participants completed a range of demographic variables as part of the King’s Centre for Military Health Research Health and Wellbeing Cohort Study [19], from which participants were screened into the clinical interview study. Gender was assessed using a binary approach (e.g., man/woman). Additionally, participants were asked about their current serving status (i.e., serving or ex-serving) during the interview study. For the purpose of this study, we included data on gender, age at interview, serving status at interview, engagement at screening, service branch at screening, and rank at screening.

### Data analysis plan

All analyses were conducted in Stata version 17. The analysis plan was pre-registered (https://osf.io/zntdu/?view_only=3efd0cef07664fc693d96cf95efa4bf3) and all deviations from this planned analysis are explicitly outlined below.

First, men and women were compared on demographic variables to identify potential confounding variables Although it was initially planned to examine relationship status, number of children, and length of service as potential confounding variables, these variables were only available in the original cohort study, and so it was decided not to include these, as they could have changed. Additionally, in line with previous studies, we decided to include rank as a potential confounding variable [18]. Differences were examined using Pearson’s chi-squared test to account for sampling weights. Secondly, men and women were compared on the variables of interest (stigma and access to care subscales, self-stigma) using univariate analyses. Lastly, using regression analysis, men and women were compared on the variables of interest adjusting for demographic variables that were identified as differing significantly between men and women in the first step of the analyses, as well as current mental health disorders based on screening measures. No issues with multicollinearity were detected.

All analyses were adjusted for sampling weights using the svy command. We had originally planned to deal with missing data using the full information maximum likelihood framework through the sem-suite in Stata. However, as no data was missing on the variables of interest (i.e., stigma variables, access to care, and gender), we employed a regression framework instead.

## Results

### Sample characteristics

Men (*n* = 1229, 84.9%) and women (*n* = 219, 15.1%) differed on several demographic aspects. Compared to women, men were significantly older (*F*(1.99, 2885.87) = 12.26, *p* < 0.001), more likely to be ex-serving (*F*(1, 1447) = 11.69, *p* < 0.001), more likely to be Regulars (*F*(1, 1447) = 9.69, *p* = 0.002), and more likely to have a non-commissioned officer rank (*F*(2, 2888.74) = 3.04, *p* = 0.048). No differences were observed in service branch (*F*(2, 2893.56) = 1.58, *p* = 0.207). As such, age, serving status, engagement type and rank were adjusted for in subsequent analyses.

In terms of current mental health, no differences were observed for probable anxiety disorder (*F*(1, 1446) = 0.90, *p* = 0.342), probable depression (*F*(1, 1446) = 0.01, *p* = 0.919), or probable PTSD (*F*(1, 1447) = 0.19, *p* = 0.659). However, men were more likely to report current alcohol misuse compared to women (*F*(1, 1447) = 17.40, *p* < 0.001) (Table 1).

**Table 1:** Demographic characteristics of study participants (*n* = 1448)

	Men (*n* = 1229)	Women (*n* = 219)	Total (*n* = 1448)
Age in years (at interview)			
< 30	136	38	174
	12.47%	19.18%	13.48%
30 to < 40	376	94	470
	30.53%	42.13%	32.27%
> 39	717	87	804
	57.00%	38.69%	54.24%
Serving status (at interview)			
Serving	648	143	791
	53.05%	65.54%	54.93%
Ex-serving	581	76	657
	46.95%	34.46%	45.07%
Engagement (pre-interview)			
Regular	1018	162	1180
	82.69%	73.74%	81.34%
Reservist	211	57	268
	17.31%	26.26%	18.66%
	46.95%	34.46%	45.07%
Service branch (pre-interview)			
Naval services	170	27	197
	13.29%	11.86%	13.08%
Army	802	135	937
	66.48%	62.69%	65.91%
RAF	257	57	314
	20.23%	25.44%	21.01%
Rank (at interview)			
NCO	762	117	879
	62.20%	53.73%	60.93%
Officer	309	71	380
	24.01%	31.15%	25.08%
Other ranks	158	31	189
	13.79%	15.12%	13.99%
Probable anxiety disorder (at interview)	216	44	260
	17.80%	20.52%	18.21%
Probable depression (at interview)	93	17	110
	7.72%	7.92%	7.75%
Probable PTSD (at interview)	107	17	124
	8.79%	7.87%	8.65%
Probable alcohol misuse (at interview)	249	18	267
	20.38%	8.36%	18.57%

Unweighted counts and weighted percentages are presented

Total may not add up due to weighting

*RAF* Royal Air Force,* NCO* non-commissioned officer, *PTSD *Post-traumatic stress disorder

### Gender differences

No significant gender differences were found for any of the stigma domains or access to care in both the unadjusted and adjusted analyses, for either mean or IRT scores. The full results of the adjusted analyses are displayed in Table 2.

**Table 2:** Results for univariate and multivariate regression analyses of stigma and access to care variables

	Access to care	Self-stigma (treatment)	Self-stigma (mental illness)	Public stigma (treatment)
Univariate analyses				
Mean scores				
Male	Reference			
Female	0.06 [− 0.03; 0.15]	0.04 [− 0.09; 0.17]	0.06 [− 0.08; 0.20]	0.05 [− 0.04; 0.14]
IRT scores				
Male	Reference			
Female	0.09 [− 0.03; 0.20]	0.08 [− 0.06; 0.21]	0.07 [− 0.06; 0.21]	0.09 [− 0.04; 0.21]
Multivariate analyses				
Mean scores				
Male	Reference			
Female	0.05 [− 0.04; 0.14]	0.05 [− 0.08; 0.18]	0.04 [− 0.10; 0.17]	0.06 [− 0.03; 0.15]
IRT scores				
Male	Reference			
Female	0.08 [− 0.04; 0.20]	0.09 [− 0.05; 0.22]	0.05 [− 0.08; 0.18]	0.09 [− 0.03; 0.22]

Regression coefficients and 95% confidence intervals are displayed

Multivariate analyses adjusted for age, service status, rank, engagement, probable depression, probable anxiety disorder, probable PTSD, and probable alcohol misuse

*IRT* item response theory

### Exploratory analyses

In addition to the pre-registered analyses, we considered whether gender differences would vary by serving status and whether there would be significant differences on individual scale items as opposed to the overall scores. We first considered an interaction between gender and serving status. The interaction term was not significant for any of the four domains (*p*s > 0.05) in either the unadjusted or adjusted analyses.

**Fig. 1:**
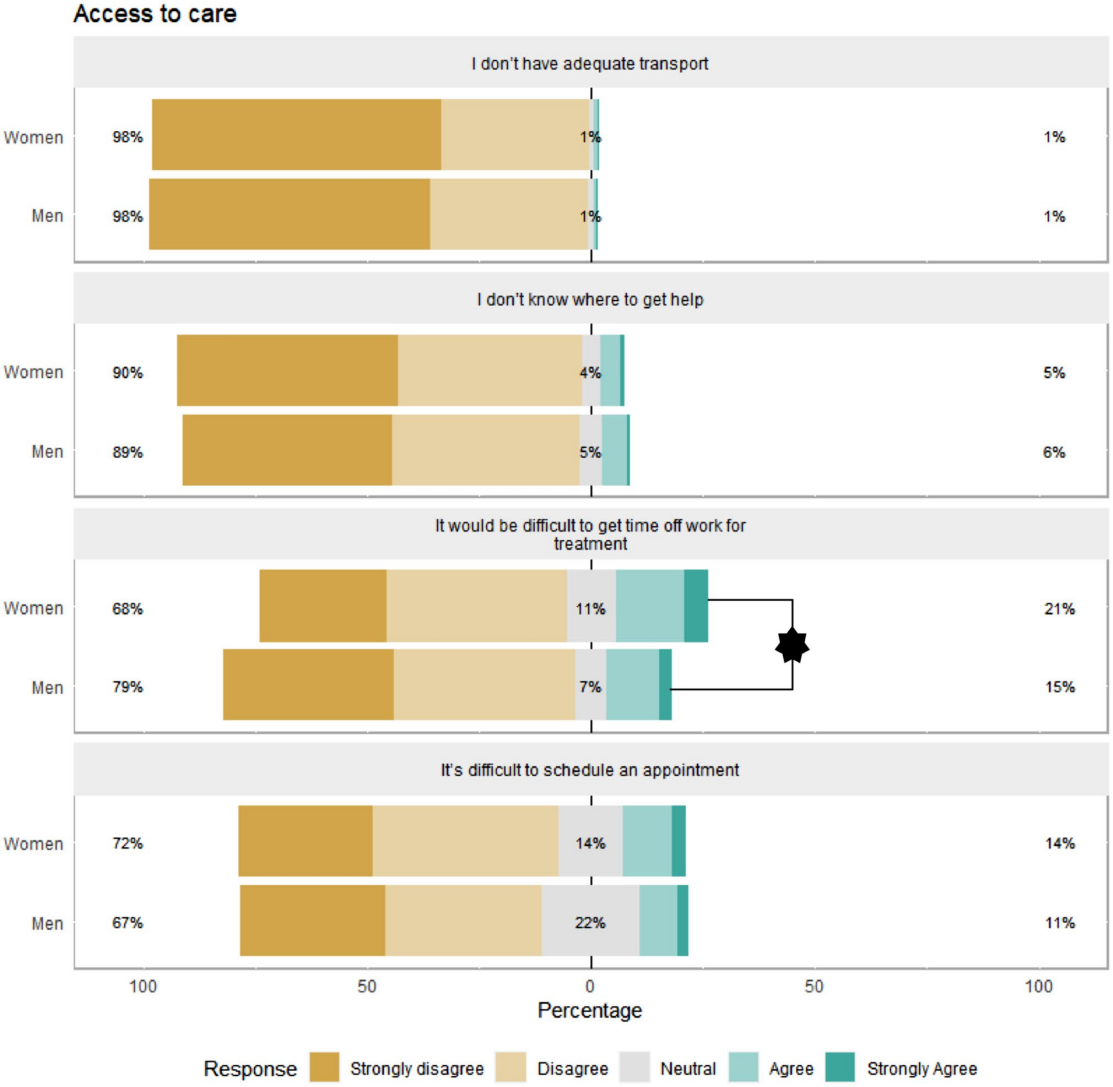
Gender differences in individual access to care barriers. Percentages on the left and right-hand sides are combined percentages for the disagree/strongly disagree and agree/strongly agree categories. *p < 0.01

**Fig. 2:**
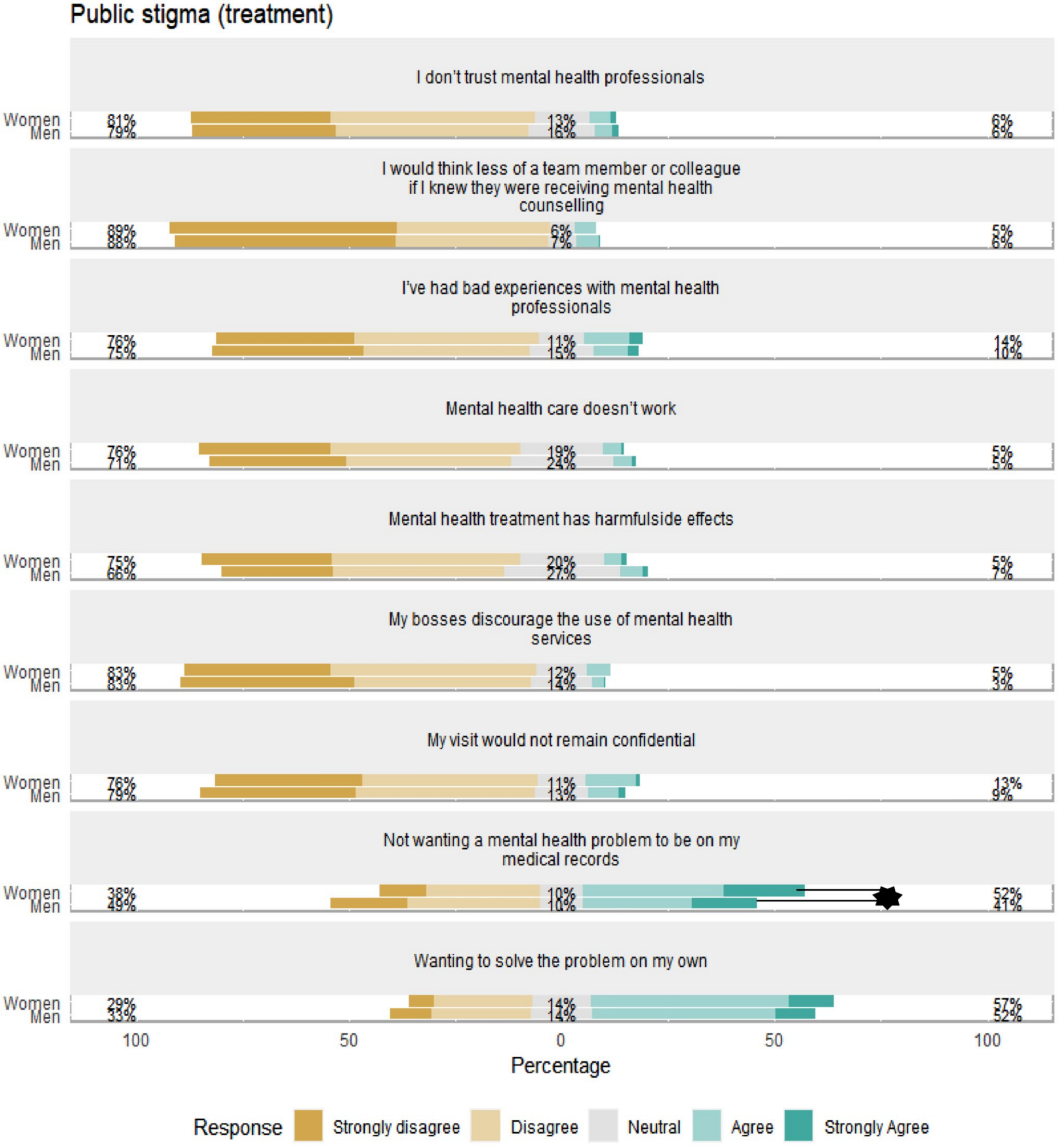
Gender differences in individual public stigma regarding treatment. Percentages on the left and right-hand sides are combined percentages for the disagree/strongly disagree and agree/strongly agree categories. *p < 0.01

**Fig. 3:**
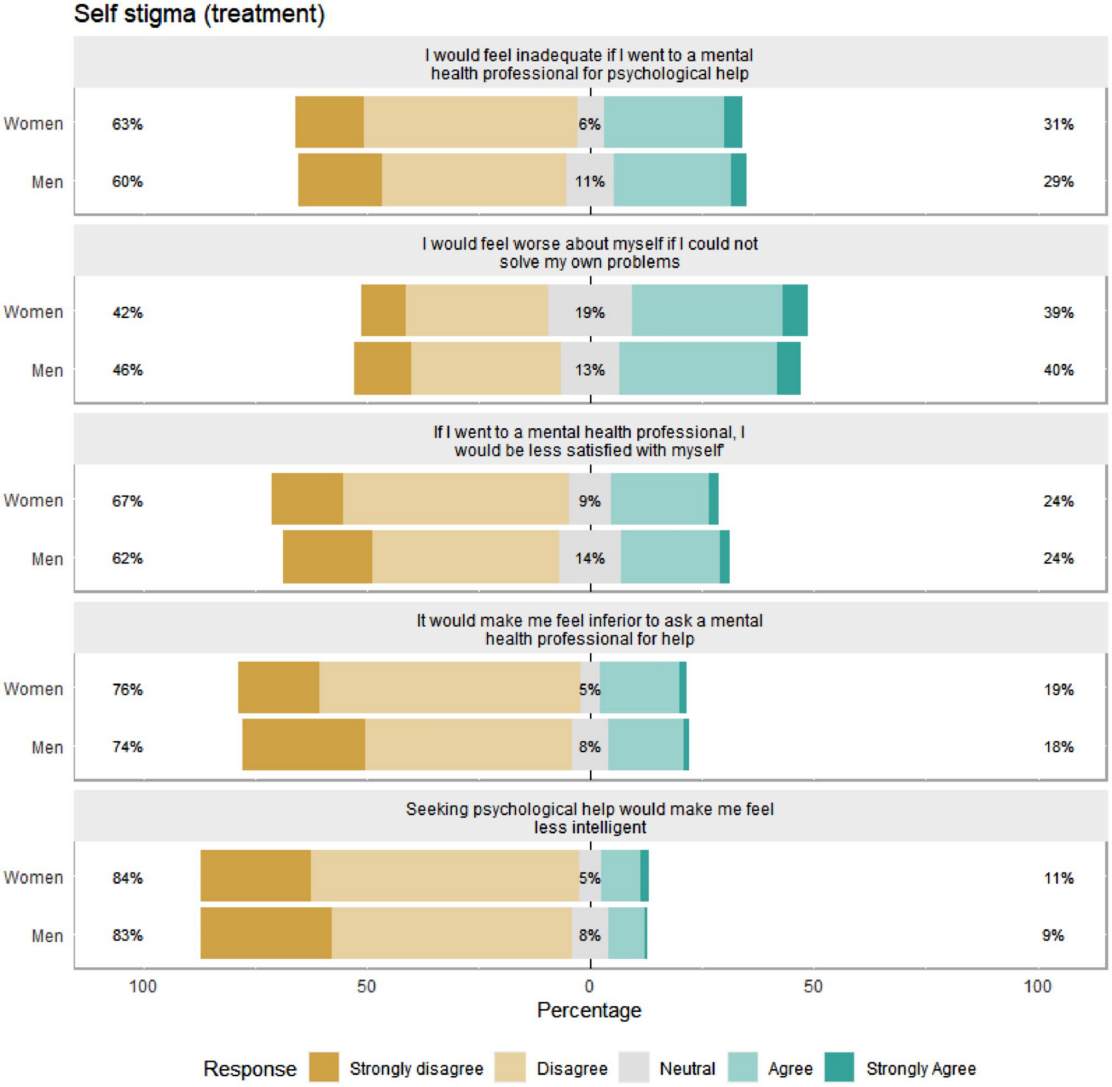
Gender differences in self-stigma regarding treatment. Percentages on the left and right-hand sides are combined percentages for the disagree/strongly disagree and agree/strongly agree categories

**Fig. 4:**
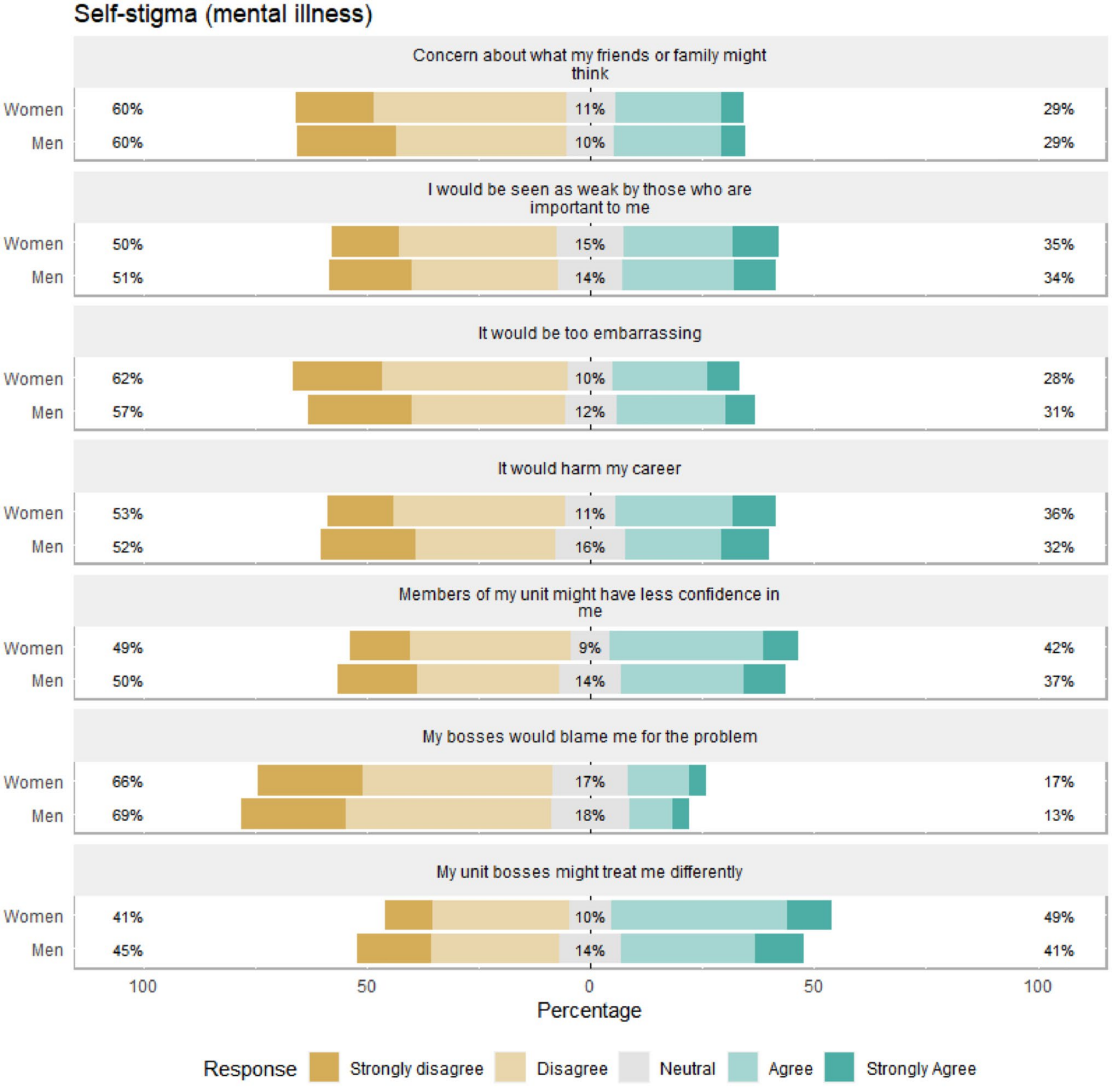
Gender differences in self-stigma regarding mental health problems. Percentages on the left and right-hand sides are combined percentages for the disagree/strongly disagree and agree/strongly agree categories

Secondly, we examined gender differences on the individual items of the stigma scales using ordered logistic regression analyses. Given the large number of comparisons made, we set the critical alpha to *p* < 0.01 for these analyses. Significant differences were observed for the items “*It would be difficult to get time off work for treatment*” and “*Not wanting a mental health problem to be on my medical records*”, which women endorsed more compared to men (21% vs 15%, OR = 1.59 [1.22, 2.08]; 52% vs 41%, OR = 1.53 [1.19, 1.96], respectively). This difference remained significant when adjusting for covariates. see Figs. 1, 2, 3 and 4.

## Discussion

The current study examined potential gender differences in stigma and access to care among serving and ex-serving members of the UK Armed Forces. Overall, men and women reported similar levels of stigma and access to care, with no significant differences detected. The highest scores for both men and women were observed in the two self-stigma domains, which encapsulate internalised attitudes and beliefs about mental illness and treatment.

Findings are in line with emerging research among both US and UK veterans [16, 17], extending these findings to the serving population of the Armed Forces. These findings add to existing literature on help-seeking among military personnel, which suggest that despite differences in rates of help-seeking whereby women are generally more likely to seek help compared to men [18], there are no differences in attitudinal or structural barriers to care. Given that stigma and access to care are often noted as key deterrents to help-seeking [1], this research suggests that other factors may explain gender differences in help-seeking. Future research should examine potential gender differences in recognition of mental health problems, as well as other factors that might explain gender differences in help-seeking, such as perceived need [30].

Results from the exploratory analyses showed that there were only two barriers in which gender differences were apparent: *difficulty getting time off work* and *concerns about mental illness on medical records*. For both barriers, women were more likely to report these than men. This is somewhat in line with findings from the general population, whereby women report greater structural barriers to mental healthcare due to family responsibilities compared to men [14, 15]. It may be that women are more concerned about getting time off work for treatment as they also need to balance family responsibilities, such as childcare, outside of work hours impacting upon their availability for treatment. Additionally, qualitative research among UK veterans indicated that women with mental health problems may experience additional scrutiny as they might be perceived as ‘weak’ both due to their gender and due to mental health problems [16]. Thus, women may be more concerned about their medical records compared to men as women may have concerns regarding who has access to information about their mental health. This partially aligns with findings from gender differences in veteran-specific services, which are more likely to be utilised by men compared to women [31]. However, further research should investigate these potential gender-specific barriers.

The current study has several policy implications. The lack of gender differences found in stigma and access to care suggests that anti-stigma campaigns would be beneficial for both men and women within the Armed Forces community. Furthermore, while women in the general population may experience lower levels of stigma compared to men, this does not appear to be the case for women in the Armed Forces. Thus, civilian service providers should be mindful that women in the Armed Forces may experience higher levels of stigma compared to women in the general population, which may impact their help-seeking attitudes. Similarly, veteran-specific services, who are currently less likely to be sought out by women who have previously served in the Armed Forces [17, 31], could address concerns about privacy and medical record keeping, encouraging more women to utilise these services. Nevertheless, most importantly women should be able to access the support they need and deserve whether it is veteran specific or not.

While the study had several strengths, some limitations should be noted. Importantly, the current study did not examine gender-specific barriers to care that may have impacted women. In a recent qualitative study among UK veterans, women reported a lack of recognition and understanding among mental healthcare providers of the issues women in the military face as a specific barrier [16]. It may be that these gender-specific barriers to care contribute towards the difference in help-seeking between men and women. Secondly, the current study examined gender using a binary approach. Gender-diverse members of the Armed Forces may face unique structural barriers compared to both men and women [32], and future research should employ more inclusive measures of gender. Thirdly, while all measures used in the current study to examine stigma have been used and validated within military samples, certain aspects of stigma may be gender-specific and not captured within the current measures. Fourthly, while we controlled for probable mental health problems in all analyses, we were unable to examine potential gender differences among individuals with a probable mental health problem (i.e., individuals most in need for professional support). As previous research found higher rates of stigma among members of the Armed Forces with a probable mental health problem [24], future research should examine whether this holds true by gender, or whether gender differences exist within individuals with current mental health difficulties. Lastly, while the current study assessed structural and attitudinal measures of stigma, we did not have a measure of public stigma of mental illness which may further develop our understanding of how gender may impact mental health stigma.

In conclusion, the current study adds to emerging research to suggest that contrary to findings among the general population, there are no gender differences in the reporting of stigma or access to care in relation to mental healthcare among serving and ex-serving members of the UK Armed Forces. These findings have implications for both anti-stigma campaigns and provide further context for those providing mental health treatment services for men and women in the UK Armed Forces community.

## Data Availability

Data are available upon reasonable request from the senior author (S. A. M. Stevelink).
